# Guided Tissue Regeneration of Periodontal Infrabony Defects with Frozen Radiation-Sterilized Allogenic Bone Graft Versus Deproteinized Bovine Bone Mineral: 5-Year Outcomes of RCT

**DOI:** 10.3390/jfb16030095

**Published:** 2025-03-10

**Authors:** Bartłomiej Górski, Aniela Brodzikowska, Kacper Nijakowski, Mariano Sanz

**Affiliations:** 1 Department of Periodontal and Oral Mucosa Diseases, Medical University of Warsaw, 02-097 Warsaw, Poland; 2 Department of Conservative Dentistry, Medical University of Warsaw, 02-097 Warsaw, Poland; 3 Department of Conservative Dentistry and Endodontics, Poznan University of Medical Sciences, 60-812 Poznan, Poland; 4 ETEP Research Group, Department of Dental Clinical Specialties, Faculty of Odontology, University Complutense of Madrid, 28040 Madrid, Spain

**Keywords:** allograft, bone graft, guided tissue regeneration, intra-bony, osseous defect, periodontal, periodontitis, regeneration

## Abstract

The aim of this study was to compare the efficacy of the guided tissue regeneration (GTR) of periodontal infrabony defects using the frozen radiation-sterilized allogenic bone graft (FRSABG) versus deproteinized bovine bone mineral (DBBM) 5 years after treatment. The association between patients’ compliance and periodontitis recurrence with 5-year outcomes was also evaluated. Thirty infrabony defects in 15 stage III/IV periodontitis patients were randomly allocated to the FRSBAG group (tests) or the DBBM group (controls). Between 1 and 5 years, one patient was lost to follow-up and one tooth was extracted due to root fracture. No tooth was extracted for periodontal reasons. Consequently, 13 teeth in test sites and 14 teeth in control sites were available for the 5-year analysis. The clinical attachment level gain (CAL-G, primary outcome), probing pocket depth (PPD), radiographic defect depth (DD), and linear defect fill (LDF) were examined at baseline and 5 years post-surgically. Both groups showed statistically significant improvements in all evaluated clinical and radiographic parameters at 5 years, with insignificant intergroup differences. CAL-Gs were 4.46 ± 2.07 mm in the FRSBAG group, and 3.86 ± 1.88 mm in the DBBM group (*p* = 0.5442). In six (43%) patients, we observed periodontitis recurrence, among whom two (33.33%) participated regularly in supportive periodontal care (SPC) and the other four (66.7%) did not take part in SPC. A regression analysis revealed that periodontitis recurrence was a significant predictor of CAL loss and DD increase. FRSBAG and DBBM were both equally effective 5 years after the GTR of infrabony defects. Within the limitations of the present study, its outcomes advocate that both grafts may be considered as a viable option based on patient preferences and clinical considerations.

## 1. Introduction

Periodontitis is associated with the progressive destruction of tooth-supporting tissues. Its symptoms include clinical attachment loss (CAL), the presence of periodontal pockets, gingival inflammation measured as bleeding on probing (BOP), and radiographically assessed alveolar bone loss [1]. Angular infrabony defects, if left untreated, demonstrated an increased frequency of progressive bone loss [2]. Moreover, the risk of tooth loss increased with the increasing depth of the infrabony lesion. Twenty-two percent of teeth with angular defects of degree 1 (2 mm) were lost over a 10-year period, as compared to 46% and 68% of teeth with angular defects of degrees 2 and 3 (2.5–4 and >4.5 mm), respectively. In another study, angular infrabony defects together with periodontal pockets ≥ 5 mm were significant predictive factors related to the progression of periodontitis in patients who participated in regular supportive periodontal care [3].

In order to reduce probing pocket depths and gingival inflammation, periodontal therapy should be carried out in line with a stepwise approach [4,5]. During the first step of therapy, the adequate patient’s oral hygiene routines and risk factor control should be achieved. Then, during the second step of therapy, the subgingival instrumentation to remove subgingival biofilm and calculus is performed. In sites which do not respond appropriately to the second step of therapy (presence of pockets ≥ 4 mm with BOP or existence of deep pockets ≥ 6 mm), the third step should be considered. In the case of residual pockets associated with infrabony defects deeper than 3 mm, regenerative surgical therapy is recommended to improve tooth prognosis [4].

Currently, guided tissue regeneration (GTR) using resorbable membranes with or without bone-derived grafts might be considered the standard of care for treating angular infrabony defects, especially in non-supporting lesions [4,6]. The placement of a barrier membrane promotes bone formation because it does not allow the downward migration of connective and epithelial tissues inside the defect [7]. The mean reported benefit of CAL gain was 1.5 mm (equivalent to a 90% improvement) for GTR combined with bone-derived grafts, as opposed to open-flap debridement (OFD) [4]. The choice of graft material (either autografts or allogenic or xenogeneic bone substitutes) should be based on defect configuration and an understanding of regenerative biology and technology [7]. Among the wide range of grafts used today, there is currently evidence of true periodontal regeneration for demineralized freeze-dried bone allograft (DFDBA) and for deproteinized bovine bone mineral (DBBM) [8,9]. DBBM promoted periodontal regeneration mostly through its osteoconductive potential, wound stability, and space provision [10]. Allogenic grafts, on the other hand, can have both osteoconductive and osteoinductive properties [11]. However, allogenic graft potential may differ greatly depending on the donor, development process, and storage techniques [12,13]. Among available banked allografts are fresh-frozen (FFBA), freeze-dried bone allografts (FDBA), and DFDBA, among which FDBA and DFDBA are the allograft forms most used [14]. Due to the strict tissue banking guidelines and processing protocols performed in a GMP-Class A environment, the risk of primary infections and antigenicity is reasonably low [15]. Moreover, allograft may be purchased in a large amount from tissue banks and has a long shelf life because of vacuum storage conditions [16]. Additionally, the customization of FDBA allows for the creation of patient-specific grafts that can be stored and used at room temperature [14]. Another allograft’s processing has led to obtaining frozen, radiation-sterilized, allogenic bone graft (FRSABG), which showed enhanced osteoinductive properties and faster remodeling than lyophilized irradiated allogenic bone graft [17]. It should be highlighted, however, that high doses of ionizing radiation may bring about numerous chemical and physical changes that in turn can undermine the biological quality of tissue allografts [18].

In our previous work, we compared the potential of GTR with FRSABG as a bone replacement graft with GTR + DBBM in the management of periodontal infrabony defects [19]. Both grafts yielded successful and comparable 12-month clinical outcomes, but the use of FRSABG significantly enhanced probing pocket depth reduction (PPD-R) and linear defect fill (LDF). These results confirmed that FRSABG may be a suitable biomaterial for the GTR of infrabony defects in terms of 12-month stability. The question remains whether the improved 12-month clinical outcomes can be maintained in the medium and long term. Several studies provided evidence that regenerative periodontal surgery may be associated with clinically important improvements five years after the treatment of infrabony defects using different biomaterials and various approaches [20,21,22]. Regenerative therapy was even shown to have the potential to change the prognosis of severely compromised teeth by attachment loss to the apex in a 5-year follow-up [23]. To the best of our knowledge, no study evaluated the performance of FRSBAG for longer than 12 months. In addition to this, the recurrence of periodontitis in patients in supportive periodontal care (SPC), the impact of patients’ compliance, and periodontitis recurrence on long-term GTR outcomes were also not verified. Despite the known advantages of either allogenic or xenogeneic grafts in periodontal regenerative treatment, there is a lack of comparative studies evaluating their effectiveness in the long term. Given the limited evidence evaluating FRSABG, further studies are necessary to optimize the outcomes of the regenerative therapies of infrabony defects. The objective of this randomized controlled trial (RCT) was to compare the medium-term clinical and radiographic results of GTR with FRSBAG versus GTR with DBBM in the treatment of periodontal infrabony defects in patients diagnosed with stage III or IV periodontitis. The null hypothesis was that 5-year CAL gain in the FRSBAG group would be no better than in the DBBM group 5 years after surgery.

## 2. Materials and Methods

### 2.1. Study Design

This is a 5-year follow-up of a split-mouth, double-blinded RCT. The design of the original trial has been reported along with the 1-year results and the details of randomization and allocation concealment [15]. It was approved by the Bioethics Committee of the Medical University of Warsaw (KB/209/2017) and registered on ClinicalTrials.gov (NCT03340012). All patients gave informed consent to participate in this RCT. This trial was conducted in line with the Helsinki Declaration of 1975, as revised in Tokyo in 2013.

### 2.2. Study Population

Patients were selected from those attending the Department of Periodontology of the Medical University of Warsaw between June 2018 and December 2019. Only non-smoking adults aged 18 years old and older, systematically healthy, and with high compliance were recruited. They had to be diagnosed with stage III or IV periodontitis and had at the minimum two teeth with a PPD ≥ 6 mm, a CAL ≥ 6 mm, and an infrabony radiographic defect depth (DD) ≥ 4 mm, as evaluated on a digital periapical radiograph. General exclusion criteria were as follows: pregnancy or lactation, smoking, systemic diseases that would adversely influence wound healing, and systemic medications interfering with post-operative wound healing. Local exclusion criteria for selected teeth were as follows: III-degree mobility, furcation involvement, pulpal pathology, or inadequate endodontic treatment.

Three months after the non-surgical periodontal treatment consisting of scaling and root planing, 15 patients (9 women and 6 men) with a mean age of 38.7 ± 7.6 years were enlisted in this RCT. They had to demonstrate a full-mouth plaque score (FMPS) ≤ 20% and a full-mouth bleeding score (FMBS) ≤ 20%. In each subject, two teeth with a PPD ≥ 6 mm, a CAL ≥ 6 mm, and a DD ≥ 4 mm were identified. The sample size was determined a priori at 15 patients (30 defects per arm) based on the pilot nature of this study. The tooth population comprised 8 upper incisors and canines, 10 premolars, and 12 molars. Fifteen teeth were located in the maxilla.

**Figure 1:**
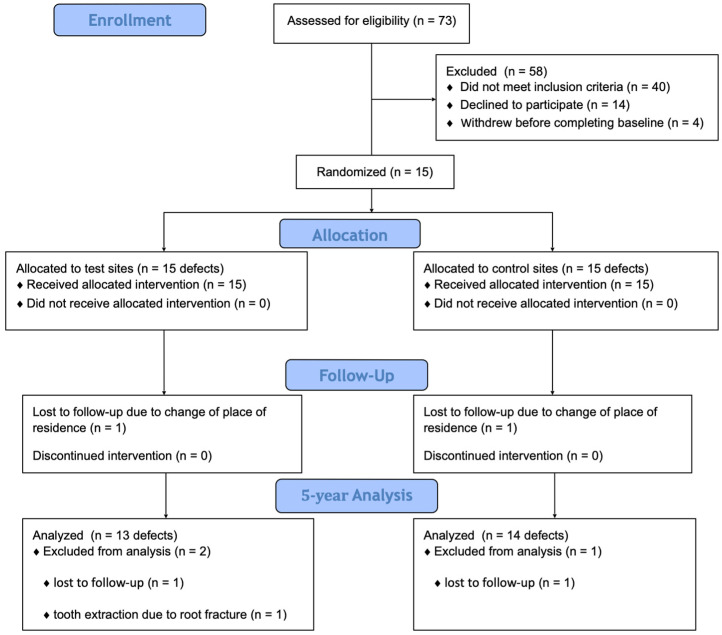
Consort diagram presenting the study outline.

The study outline is presented in Figure 1.

### 2.3. Clinical Measurements

The clinical parameters were evaluated at baseline, 1 year, and 5 years post-operatively by a calibrated examiner who was blinded to treatment. Calibration exercise was executed in five non-study stage III or IV periodontitis patients. Full-mouth PPD and CAL were measured twice within 24 h. Calibration was accepted when ≥90% of the measurements could be reproduced with an intra-examiner agreement within 1.0 mm in ≥90%.

The following clinical measurements were recorded:

- CAL: distance between the cementoenamel junction (CEJ) or between the apical margin of the crown/restoration and the pocket base;
- PPD: distance between the gingival margin and the pocket base;
- Gingival recession (GR): distance between the CEJ and gingival margin;
- FMPS: the percentage of teeth surfaces with the presence of plaque [24]; all surfaces of all teeth were scored;
- FMBS: the percentage of pockets bleeding after probing [25]; all surfaces of all teeth were scored.

CAL, PPD, and GR were recorded at six sites in each affected tooth with a periodontal probe (PCP UNC 15; Hu-Friedy, Chicago, IL, USA) and rounded off to the nearest millimeter. The sites with the greatest pre-surgical CAL values were used for the statistical analysis.

The primary outcome variable was CAL gain (CAL-G). The secondary outcomes were PPD-R and the changes in GR. The analyzed metrics were calculated as follows:

- CAL-G1 = CAL0–CAL1 (after 1 year);
- CAL-G5 = CAL0–CAL5 (after 5 years);
- PPD-R1 = PPD0–PPD1 (after 1 year);
- PPD-R5 = PPD0– PPD5 (after 5 years);
- GR1 = GR0–GR1 (after 1 year);
- GR5 = GR0–GR5 (after 5 years).

### 2.4. Radiographic Measurements

Digital periapical radiographs were carried out with the parallel long-cone technique using a customized film-holder device at baseline, 1 year, and 5 years, as previously described [19]. The following measures were performed by an independent calibrated clinician who was blinded to treatment. An intra-examiner calibration exercise was carried out by evaluating 10 non-study-related X-rays prior to the commencement of the study.

Two lines were drawn, first through the tooth axis (AUX1) and second (AUX2) from the alveolar bone crest perpendicular to the AUX1. The following radiological measurements were recorded:

- CEJ-BD: the distance from the CEJ to the bottom of the defect;
- CEJ-AC: the distance from the CEJ to the interdental bone crest;
- DD: the distance from the spot where the AUX2 crossed the CEJ–BD line to the BD;
- Radiographic angle: the angle between the AUX1 and AUX2 of the treated tooth.

The radiographic DD, LDF, and percentage of defect fill (%DF) were secondary outcomes. The analyzed metrics were calculated as follows:

- LDF1 = CEJ-BD1-CEJBD0 (after 1 year);
- LDF5 = CEJ-BD5-CEJ-BD0 (after 5 years);
- %DF1 = LDF1/DD0 (after 1 year);
- %DF5 = LDF5/DD0 (after 5 years).

### 2.5. Randomization and Allocation Concealment

Randomization was performed with a computer-generated randomization list by a statistician who did not participate in this study. The allocation was kept hidden in sealed envelopes. The envelopes were opened prior to the surgery and the treatment allocation was disclosed to the surgeon. Patients were blinded to allocation.

### 2.6. Surgical Procedures and Intra-Surgical Measurements

All 15 patients were treated by the same experienced clinician (BG) as previously reported [19]. In brief, after local anesthesia, the defect-associated interdental papilla was surgically approached with the modified papilla preservation technique (MPPT for an interdental space width > 2 mm) or the simplified papilla preservation flap (SPPF for an interdental space width ≤ 2 mm) [26,27]. A full-thickness envelope mucoperiosteal flap was prepared, giving access to the infrabony lesion. The granulation tissue was carefully removed, and the root surfaces were planed.

Intra-surgical measurements were recorded:

- Defect depth: the distance from the bottom of the defect to the most coronal point of the bony walls surrounding the defect;
- Defect width: the distance between the most coronal part of the bony walls surrounding the defect and the root surface;
- Defect morphology: defects were divided into one-wall, two-wall, and three-wall defects based on the number of present walls.

In the next step, the allocation concealment was revealed to the surgeon. In each patient, the infrabony defect was filled with FRSABG in the test site or DBBM (Bio-Oss, Geistlich Biomaterials, Princeton, NJ, USA) in the control sites. For both sites, a porcine-derived collagen membrane (Bio-Gide, Geistlich Biomaterials) was adjusted to the morphology of the defect. Subsequently, flaps were sutured without tension using apical horizontal mattress sutures (Seralon 5/0 15 mm 3/8, Serag-Wiessner GmbH & Co., Naila, Germany) and coronal vertical mattress sutures (Seralon 6/0 12 mm 3/8).

### 2.7. Post-Surgical Period and Supportive Periodontal Care

Patients were instructed to rinse three times per day with 0.2% chlorhexidine and to avoid brushing or chewing for two weeks. They were asked to take non-steroidal anti-inflammatory drugs (600 mg of ibuprofen twice a day for 2 days) and antibiotics (1 g of amoxicillin clavulanic acid twice a day for 1 week). The sutures were removed after 2 weeks. The follow-up visits were set every 2 weeks for the first 3 months and every 3 months for 1 year to limit the confounding effect of suboptimal plaque control on healing.

After 1 year, subjects were scheduled for SPC with recall intervals from 3 to 6 months, and the involvement in SPC was registered. The recurrence of periodontitis was recognized when the following occurred:

- BOP was observed at >10% of sites, and
- sites with a PPD ≥ 4 mm which exhibited bleeding on probing were present [4].

Periodontal therapy was provided to sites with periodontitis recurrence (PPD ≥ 4 mm with BOP).

### 2.8. Statistical Analysis

Statistical analysis was performed using Statistica 13.3 (Statsoft, Cracow, Poland). The normality of distribution for quantitative variables was assessed and confirmed by the Shapiro–Wilk test. Student’s *t*-test was used for comparing the means of the test and control groups, as well as between baseline and follow-ups. Data were presented as the means, standard deviations (SDs), and 95% confidence intervals (CIs). Multiple linear regression was applied to analyze the relationships between patient age, patient sex, tooth type, FMPS, FMBS, PPD, CAL, DD, RVG angle at 1 year, surgical modality, patients’ compliance, and the recurrence of periodontitis with PPD-G, CAL-R, and LDF 5 years post-operatively (dependent variables). Significance was set at *p* < 0.05.

Data after 1 year indicated that the difference in the CAL response of matched pairs was normally distributed with a standard deviation of 1.18. If the true difference in the mean CAL response of matched pairs was 5.54, we would need to study 3 pairs of subjects to be able to reject the null hypothesis with a probability (power) of 0.9. (based on the power calculations using PS Power and Sample Size Calculations v. 3.1.6 by William D. Dupont and Walton D. Plummer).

## 3. Results

### 3.1. Experimental Population

The details of the baseline data are shown in Table 1, and there was no statistically significant difference at baseline between the two groups. No adverse events were recorded during the follow-up period, but membrane exposure was observed in three of the test sites and three of the control sites 2 weeks post-operatively. Between 1 and 5 years, one patient dropped out due to a change in the place of residence. In one patient, one tooth was extracted because of a root fracture (test site). No tooth had to be extracted for periodontal reasons. Consequently, 13 teeth in test sites and 14 teeth in control sites were available for the 5-year analysis.

Among fourteen patients, eight (57%) took part in regular professional SPT, and six (43%) received no SPC. Six (43%) patients exhibited the recurrence of periodontitis, among whom two (33.33%) were involved in regular SPC, whereas the other four (66.7%) did not.

### 3.2. Clinical and Radiographic Outcomes

Table 2 depicts the CAL, PPD, GR, and radiographic changes at 1 and 5 years. Compared to baseline values, the FRSBAG group demonstrated a significant CAL gain of 5.54 ± 1.18 mm, while the DBBM group showed a significant CAL-G of 4.54 ± 1.11 mm after 1 year with an insignificant intergroup difference (*p* = 0.0891). After 5 years, the CAL-G was 4.46 ± 2.07 mm and 3.86 ± 1.88 mm, respectively, with insignificant intragroup and intergroup differences.

Likewise, when compared to baseline, a significant PPD-R was observed in the test group of 4.66 ± 1.24 mm, and 3.54 ± 1.90 mm and in the control group of 3.57 ± 1.1 mm, and 3.00 ± 1.80 mm at 1 and 5 years, respectively, with a significant intergroup difference (*p* = 0.0190) after 1 year, and an insignificant intergroup difference (*p* = 0.4560) after 5 years.

For GR, no significant intragroup or intergroup differences were observed at baseline, at 1 year, and at 5 years.

**Figure 2:**
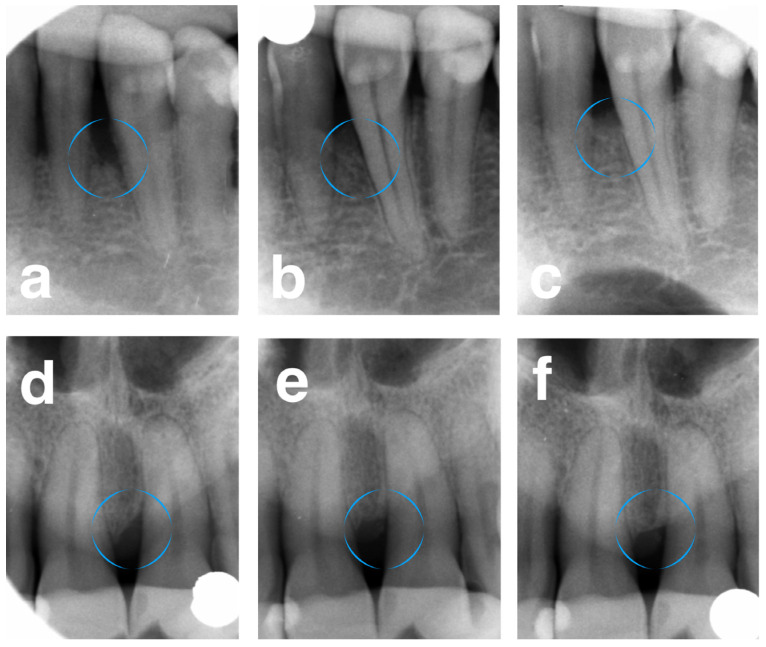
(**a**) Pre-operative X-ray of the infrabony defect on the mesial surface of tooth 33 (test site). (**b**) X-ray 1 year post-operatively. (**c**) X-ray 5 years post-operatively. (**d**) Pre-operative X-ray of the infrabony defect on the mesial surface of tooth 21 (control site). (**e**) X-ray 1 year post-operatively. (**f**) X-ray 5 years post-operatively. Circular outlines highlight infrabony defects.

For DD, the FRSBAG group showed significant improvement from 5.89 ± 1.23 to 0.66 ± 0.45 at 1 year and to 0.98 ± 0.62 at 5 years, versus the DBBM group, which demonstrated 5.32 ± 1.84, 0.92 ± 0.61, and 1.14 ± 0.54, pre-operatively, after 1 year, and after 5 years, respectively. The significant intergroup difference was observed at 1 year (*p* = 0.0334), while insignificant differences were seen at baseline and after 5 years. Similar patterns were demonstrated for LDF and %DF (Figure 2).

Five defects (38.46%) in the FRSBAG group and five defects (35.71%) in the DBBM group showed a PPD ≤ 3 mm (Table 3). One lesion (7.70%) treated with FRSBAG and two lesions (14.29%) treated with DBBM had PPDs ≥ 6 mm. The remaining defects, seven in the test group (53.84%) and seven in the control group (50%), had PPDs of 4 or 5 mm, respectively. By the same token, a PPD ≤ 5 mm was achieved in the case of 92.3% of FRSBAG-treated sites and 85.71% of DBBM-treated sites.

### 3.3. Regression Analysis

A statistically significant association between 5-year CAL and 5-year LDF and periodontitis recurrence was found in regression analysis (Table 4, Table 5 and Table 6). In terms of CAL-G, 80% of the variability might be described by the regression model (R^2^ = 0.8011), and in terms of LDF, 79% the variability might be described by the regression model (R^2^ = 0.7919). For FMPS and FMBS measurements, all surfaces of all teeth were scored, whereas PPD, CAL, and GR were site-specific.

## 4. Discussion

Even though a one-year follow-up is accepted as a minimal timeframe to draw conclusions in trials on the periodontal regenerative therapy of infrabony defects, longer follow-up observations give a more comprehensive view. In this RCT, statistically significant improvements in all evaluated parameters were noted at 5 years in both FRSBAG- and DBBM-treated lesions without statistically significant differences between groups. The 5-year CAL-G was 4.46 mm and 3.86 mm for the test and control sites, respectively, with insignificant intergroup differences; hence, the null hypothesis was not rejected. FRSBAG provided comparable PPD-R and a similar amount of bone fill with respect to DBBM. The majority of lesions (more than 90% in the test group and 85% in the control group) achieved a 5-year PPD ≤ 5 mm. All things considered, the two grafts were similarly efficacious in the GTR of infrabony defects in stage III/IV periodontitis. We also found that periodontitis recurrence was a significant predictor of 5-year CAL loss and DD increase. To the best of our knowledge, this is the first study on 5-year results following GTR with FRSBAG used as a bone replacement. Based on these observations, FRSBAG might be recommended as a feasible graft, and an alternative to DBBM in the GTR of periodontal infrabony defects. However, several factors, such as the qualities of the graft material, ease of handling, the patient’s treatment objectives, as well as the surgeon’s preferences, might be deciding factors for choosing a particular bone graft.

In a recent systematic review and meta-analysis, Nibali et al. [28] concluded that GTR provided an adjunctive benefit in terms of CAL-G (1.15 mm) and PPD-R (1.24 mm) with respect to OFD at 12-month follow-up. The addition of DBBM further improved GTR outcomes (CAL-G: 1.5 mm and PPD-R: 1.13 mm). The clinical results obtained after treatment were maintained for a period of 5 years [29,30]. The mean PPD and CAL measured 5.6 mm and 9.1 mm, respectively [29]. The achieved CAL-G of 2.09 mm was stable after at least 5 years of SPC [28]. Among available allografts, FDBA and DFDBA have been widely evaluated and are frequently used in the GTR of infrabony defects in stark contrast to radiation-sterilized allografts. According to the best of our knowledge, FRSBAG has not been exploited for the GTR of infrabony defects. When DBBM was compared to DFDBA in the treatment of infrabony defects, no statistical difference was found 6 months post-operatively [31]. The DBBM group showed a CAL-G of 3.6 mm, a PPD-R of 3.0 mm, and a %BF of 55.8%, whereas the DFDBA group achieved 2.6 mm, 2.0 mm, and 46.8%, respectively. In another RCT, the use of GTR + DFDBA significantly improved all clinical parameters in comparison to GTR alone at 6-month examination [32]. Another recent study evaluated the effect of injectable platelet-rich fibrin (I-PRF) together with DFDBA versus DFDBA alone in the management of the infrabony defects of stage III periodontitis patients [33]. The CAL-G of 2.40 ± 0.70 mm and 2.50 ± 0.85 mm and the PPD-R of 3.50 ± 1.18 mm and 2.80 ± 0.42 mm were demonstrated for I-PRF + DFDBA and DFDBA at 9 months, respectively. In both groups, a significant LDF of 3.58 ± 0.66 mm and 3.89 ± 1.57 mm for I-PRF + DFDBA and DFDBA was found at 9 months. No significant intergroup differences were reported. On the other hand, a combination of DFDBA and platelet-rich fibrin (PRF) enhanced the analyzed parameters with contrast to PRF and DFDBA alone [34]. The clinical performances of DFDBA and FDBA were directly compared with each other, yielding similar outcomes [35]. A mean CAL-G of 1.7 mm was reported with DFDBA and 2.0 mm with FDBA. A mean PPD-R was 2.00 mm with both DFDBA and FDBA, and a defect fill of 1.7 mm (59%) was achieved with DFDBA and 2.4 mm (66%) with FDBA. These findings revealed no significant differences between the two materials at a minimum of 6 months post-operatively. The stability of the outcomes after GTR treated with either FDBA or solvent-dehydrated bone allograft was evaluated in 175 infrabony defects [36]. One year post-operatively, 3.55 mm of CAL-G and 3.87 of PPD-R were observed (*p* < 0.05). The Kaplan–Meier analysis revealed that 70.4% and 54.9% of the treated sites were stable after 5 and 10 years and the survival rates of the treated teeth were 85.0% and 72.7% after 5 and 10 years, respectively. The outcomes of the present RCT are in line and compare well with the outcomes presented in the above-mentioned studies. In the FRSBAG group, the 5-year CAL-G was 4.46 and the PPD-R was 3.54 mm. Unfortunately, a direct comparison between FRSBAG and different allografts (FDBA, DFDBA) was not possible, which should be addressed in future studies.

The longer follow-up period of this RCT allowed us to assess the 5-year effectiveness and safety profile of the GTR of infrabony defects treated with both grafts, which might not have been apparent during shorter in-trial periods [37]. Consequently, the benefits achieved after 12 months were maintained at 5 years for both FRSBAG and DBBM, and no side effects appeared during this period. Our study suggests that both grafts were equally effective in the GTR of infrabony defects in stage III and IV periodontitis, which may reflect similar regenerative capabilities in promoting periodontal regeneration. Reported results imply, within this RCT’s limitations and the specific patient population, that surgeons may choose between FRSBAG and DBBM depending on specific patients factors as well as clinical deliberations to customize treatments to the personalized patient’s needs. However, different disadvantages of bone graft materials should be kept in mind. Allogenic grafts may be expensive, present high quality variations in terms of reduced osteoconductivity, and can have a risk of infection due to limited or insufficient information given by the donor and the loss in growth factors during the course of manufacturing processes [38]. Xenografts on the other hand might represent a risk of zoonotic diseases transmission [39]. In addition to these hazards and risks, religious/ethical controversies may be of utmost importance in some patients [40].

Based on pairwise comparisons, the regenerative treatment was more successful compared with OFD with respect to smaller DD and greater LDF (range of MD: −4.74 to −1.20 mm) [41]. However, caution should be exercised when explaining radiographic pictures in the presence of slowly resorbable bone grafts, such as DBBM. The possible explanation for the small absorption potential of DBBM might be the modification in the hydroxyapatite structure resulting from the high-temperature processing during the manufacturing process [42]. Slowly resorbable material may occupy a major area of the newly formed bone even after a long time interval [43]. Be that as it may, the sheer presence of bone graft particles within the regenerated tissues was not detrimental to periodontal stability in the medium and long term [41].

Achieving stability after GTR is possible, but the independent outcomes are influenced by smoking and compliance with periodontal maintenance and monitoring, all of which constitute the vital prerequisites to guarantee effective and long-term stability. In a systematic review and network meta-analysis, periodontal regenerative therapy of infrabony lesions resulted in a significantly shallower PPD (range of mean differences, MD: −2.37 to −0.60 mm) and greater CAL gain (range of MD: 1.26 to 2.66 mm) [41]. Combined approaches were shown as the most effective. The average residual PPD was <5 mm. Tooth loss was less common (0.4%) with respect to OFD (2.8%) in the medium (3–5 years) and long term (5–20 years). Therefore, the major goal of periodontal regenerative treatment is to reflect decreased tooth mortality. It should be underscored though, that the results obtained in the majority of studies reported on highly motivated, mainly non-smoking patients treated in clinical settings, which provided a high standard of periodontal maintenance; thus, external validity may be restricted [44]. As a matter of fact, the “center effect” was reported to account for approximately 2 mm in CAL-G [28]. Several studies observed that some portion of the 1-year CAL-G might be lost in both the medium and long term [41]. As a matter of fact, a CAL of 1.2 mm was observed from 1 to 5 years post-operatively [45]. The patients who achieved stable sites had good oral hygiene, took part in regular SPC, and refrained from smoking. In contrast, a partial loss of the CAL-G was observed in smokers with poor oral hygiene and a lack of compliance [46,47]. When compared to the 1-year outcome, some deteriorations in clinical parameters were clearly visible in both test and control group in this RCT, with insignificant intragroup and intergroup differences. This can be attributed to the recall protocol and the reinforcement of oral hygiene at every appointment, as well as the selection criteria that included only non-smokers with low FMPS and FMBS. In our RCT, almost 60% of the patients received long-term SPC at the university clinic. Six (43%) patients showed periodontitis recurrence, among whom four (66.7%) did not regularly participate in the SPC program. The regression analysis revealed that periodontitis recurrence was a significant predictor of 5-year CAL loss and DD increase. Be that as it may, this is the first study that evaluated the recurrence of periodontitis in SPC patients and disease recurrence in the 5-year outcomes of the GTR of infrabony defects with FRSBAG and compared it to GTR with DBM used as bone graft. Subsequently, it allowed us to detect changes in the clinical and radiographic parameters that have occurred from 1 year to 5 years post-operatively. Quite similarly, patients with periodontitis recurrence had lower CAL gain and bone density gain over a period of 4 years [48]. The lack of recurrence, on the other hand, led to a 2.94 mm CAL gain and a nearly 10% bone density gain. Moreover, regular specialist-based SPC acted as a protective factor.

Ideally, after achieving the endpoints of periodontal treatment (no PPD > 4 mm with BOP or no PPD ≥ 6 mm), patients should be in regular SPC. However, even successfully treated cases will bear the increased risk of recurrence. It should also be underscored that the proposed and widely accepted endpoints of therapy may not be achievable in all sites in patients with advanced periodontitis. Sites with a PPD ≥ 4 mm which exhibit BOP may be in need of further re-treatment. The presence of a PPD > 5 mm following periodontal treatment was indeed associated with a higher risk of recurrence, defect progression, and tooth loss, irrespective of BOP, especially compared to teeth with a residual PPD ≤ 3 mm [46]. Of note, in the present RCT, the vast majority of sites in both treatment modalities achieved a PPD ≤ 5 mm, and only one tooth (7%) in the FRSBAG-treated sites and two teeth (14%) in the DBBM-treated sites were deeper than 6 mm. The comparison between the test and control groups showed similar trends in the frequency of PPD changes. In contrast to our previous work, in this 5-year observation, we adapted recently updated EFP guidelines regarding the management of stage III-IV periodontitis regarding the endpoints of the therapy [4,5]. Another study suggested that teeth with residual vertical defects (RVDs) following active periodontal treatment may serve as risk indicators for disease progression [49]. Teeth with RVDs had a two-fold higher risk of being lost due to periodontitis in contrast to contralateral teeth with comparable PPD. This might be explained by the fact that RVDs are frequently associated with irregular bone morphology, which can pave the way for residual pockets after periodontal treatment. Unfortunately, due to a limited sample size, our material did not allow more speculation into this aspect but may be helpful in better hypothesis generation for future studies. This paper may also serve as a guide for other studies focusing on the regenerative potential of FRSBAG.

The present study used a split-mouth design, thus mitigating the influence of individual variability that may differ between patients and possibly confound study results. The major limitation of this RCT is the relatively small number of evaluated defects, which may be a potential source of bias. The presented medium-term data are pilot in nature and should be confirmed in a larger sample of patients. Second, only human histological data may provide the evidence for true periodontal regeneration, as some of the graft pieces may remain encapsulated within the connective tissue. However, such studies may raise ethical concerns, provided the need for biopsy. Consequently, in this RCT, well-accepted surrogate outcomes were used [50]. It thus remains unclear whether the observed improvements were due to qualitatively (type of histological healing) or quantitatively improved outcomes (CAL-G, PPD-R). Third, as the majority of the patients received long-term SPC at the university clinic, the generalizability of the results may be limited. Similarly, patient-reported outcomes 5 years after surgery were not evaluated; therefore, more research on patients’ preferences and feedback is required. Fourth, the split-mouth design of this RCT removed potential inter-individual variability from the estimates of the outcome variables. On the other hand, the split-mouth design may be associated with the carry-across effect leading to the downward-biased effect on the variance in treatment outcomes [51]. To limit the impact of the carry-over effect, both infrabony defects enrolled in this study were treated during a single surgery. Thus, large-scale RCTs reporting relevant clinical and radiographic outcomes are necessary to provide more comprehensive evidence and to further investigate the efficiency of FRSABG in the GTR of infrabony defects. Furthermore, the cost–benefit of long-term data should also be analyzed.

## 5. Conclusions

Within the limitations of the present study, especially a small sample size, both FRSABG and DBBM proved to be equally effective in the GTR of infrabony defects in stage III and IV periodontitis 5 years after treatment. Comparable CAL-G, PPD-R, and amount of bone fill were achieved. Consequently, both grafts may be considered as an effective and suitable bone graft material used in the treatment of infrabony defects based on patient preferences and clinical considerations. Future RCTs with larger sample sizes, broader clinical settings, and extended observation periods should be carried out to shed more light on the regenerative potential of FRSABG.

## Figures and Tables

**Table 1:** Demographic of recruited patients (mean, 95% confidence interval (CI), and standard deviation (SD)).

Variables	Test Sites (*n* = 15)	Control Sites (*n* = 15)	*p*
Molars (*n*)	6	6	
Premolars (*n*)	5	5	
Incisors, canines (*n*)	4	4	
Upper teeth (*n*)	7	8	
Lower teeth (*n*)	8	7	
RVG angle (°)	23.39 (20.39–26.40) ± 5.42	26.49 (22.04–30.45) ± 7.59	0.2467
Defect depth (mm)	6.00 (4.99–7.00) ± 1.81	5.80 (4.42–7.18) ± 2.48	0.8029
Defect width (mm)	3.73 (2.97–4.50) ± 1.39	3.06 (2.53–3.60) ± 0.96	0.1372
One wall (*n)*	5	4	
Two wall (*n*)	5	6	
Three wall (*n*)	5	5	

**Table 2:** Evaluated parameters: clinical attachment level (CAL), probing pocket depth (PPD), gingival recession (GR), radiographic defect depth (DD), linear defect fill (LDF), and percentage defect fill (%DF) at baseline and after 1 and 5 years (mean, 95% CI, and SD).

	Baseline	1 Year	5 Years	*p* Baseline—1	*p* Baseline—5	*p* 1–5
CAL test (mm) CAL control *p*	8.93 (8.13–9.73) ± 1.44 8.73 (7.81–9.69) ± 1.67 0.2219	3.23 (2.69–3.65) ± 0.83 4.13 (3.25–5.02) ± 1.59 0.1182	4.23 (3.18–5.28) ± 1.74 4.57 (3.94–5.20) ± 1.09 0.5442	<0.0001 * <0.0001 *	<0.0001 * <0.0001 *	0.5531 0.4663
∆CAL test (mm) ∆CAL control *p*		5.54 (4.40–6.55) ± 1.18 4.54 (3.95–5.22) ± 1.11 0.0891	4.46 (3.21–5.71) ± 2.07 3.86 (2.77–4.94) ± 1.88 0.4330			0.5531 0.4663
PPD test (mm) PPD control *p*	7.67 (6.98–8.35) ± 1.23 7.40 (6.82–9.66) ± 1.06 0.3427	3.23 (2.69–3.65) ± 0.83 3.63 (3.21–4.06) ± 0.77 0.0429 *	3.77 (2.98–4.56) ± 1.30 4.07 (3.30–4.84) ± 1.33 0.5561	<0.0001 * <0.0001 *	<0.0001 * <0.0001 *	0.4156 0.3093
∆PPD test (mm) ∆PPD control *p*		4.66 (3.78–5.51) ± 1.24 3.57 (3.18–5.22) ± 1.11 0.0190 *	3.54 (2.39–4.69) ± 1.90 3.00 (1.96–4.04) ± 1.80 0.4560			0.4156 0.3093
GR test (mm) GR control *p*	1.21 (5.21–6.57) ± 1.23 1.47 (0.84–2.08) ± 1.12 0.7918	0.66 (0.42–0.91) ± 0.45 1.03 (0.52–1.55) ± 0.93 0.1651	0.69 (0.07–1.32) ± 1.03 0.79 (0.18–1.39) ± 1.05 0.8178	0.4981 0.2610	0.5763 0.8764	0.3870 0.4041
∆GR test (mm) ∆GR control *p*		0.22 (−0.28–0.75) ± 0.91 0.45 (0.11–0.78) ± 0.66 0.3281	0.62 (−0.02–1.725) ± 1.04 0.71 (0.10–1.33) ± 1.07 0.8101			0.3870 0.4041
DD test (mm) DD control *p*	5.89 (5.21–6.57) ± 1.23 5.32 (4.31–6.35) ± 1.84 0.2811	0.66 (0.42–0.91) ± 0.45 0.92 (0.58–1.27) ± 0.61 0.0334 *	0.98 (0.60–1.35) ± 0.62 1.14 (0.83–1.45) ± 0.54 0.4647	<0.0001 * <0.0001 *	<0.0001 * <0.0001 *	0.0050 * 0.0313 *
LDF test (mm) LDF control *p*		5.22 (4.56–5.54) ± 1.11 4.33 (3.40–5.18) ± 1.74 0.0478 *	4.91 (4.07–5.74) ± 1.38 3.93 (3.01–4.85) ± 1.59 0.1014			0.0123 * 0.0177 *
%DF test (mm) %DF control *p*		85.89 (81.29–95.11) ± 8.90 83.27 (77.61–90.37) ± 11.41 0.1091	83.06 (76.73–89.39) ± 10.47 75.78 (68.54–83.02) ± 12.54 0.1155			0.0197 * 0.0283 *

* statistically significant (*p* ≤ 0.05).

**Table 3:** Frequency distribution of PPD at baseline and 1 and 5 years post-operatively.

	Test Sites (*n* = 15)				Control Sites (*n* = 15)			
	≤3 mm	4 mm	5 mm	≥6 mm	≤3 mm	4 mm	5 mm	≥6 mm
Baseline (*n* = 30)	-	-	-	15	-	-	-	15
1 year (*n* = 29)	7	4	3	-	7	5	1	1
4 years (*n* = 27)	5	4	3	1	5	3	4	2

**Table 4:** Regression analysis with CAL-G from 1 year to 5 years post-operatively as a dependent variable. *R*^2^ = 0.8011.

Parameter	Regression Coefficient	Standard ERROR	Confidence Interval		*p*
			Lower	Upper	
Intercept					0.008 *
Sex (male vs. female)	0.191	0.236	−0.315	0.696	0.431
Age	0.160	0.142	−0.144	0.464	0.278
Surgical procedure (test vs. control)	−0.135	0.152	−0.462	0.192	0.391
Tooth type (molars vs. incisors, canines, and premolars)	0.104	0.214	−0.355	0.563	0.634
Tooth position (upper vs. lower)	−0.016	0.190	−0.424	0.391	0.933
FMPS	0.046	0.279	−0.552	0.643	0.871
FMBS	0.214	0.257	−0.336	0.765	0.418
PPD	0.255	0.206	−0.187	0.698	0.236
DD	0.214	0.186	−0.184	0.612	0.269
RVG angle	0.159	0.177	−0.220	0.537	0.383
Patients’ compliance (1 vs. 0)	−0.059	0.178	−0.442	0.323	0.745
Periodontitis recurrence (1 vs. 0)	0.828	0.164	0.477	1.180	<0.001 *

FMPS: full-mouth plaque score, FMBS: full-mouth bleeding score, and * statistically significant (*p* ≤ 0.05).

**Table 5:** Regression analysis with PPD-R (mm) from 1 year to 5 years post-operatively as a dependent variable. *R*^2^ = 0.6051.

Parameter	Regression Coefficient	Standard Error	Confidence Interval		*p*
			Lower	Upper	
Intercept					0.192
Sex (male vs. female)	−0.071	0.328	−0.7777	0.630	0.825
Age	−0.273	0.200	−0.703	0.156	0.194
Surgical procedure (test vs. control)	−0.121	0.215	−0.582	0.340	0.582
Tooth type (molars vs. incisors, canines, and premolars)	0.231	0.316	−0.447	0.910	0.476
Tooth position (upper vs. lower)	−0.244	0.389	−1.078	0.589	0.540
FMPS	0.099	0.347	−0.645	0.842	0.780
FMBS	−0.330	0.285	−0.942	0.281	0.266
CAL	−0.057	0.261	−0.617	0.504	0.832
DD	0.027	0.249	−0.506	0.561	0.914
RVG angle	−0.244	0.389	−1.078	0.589	0.540
Patients’ compliance (1 vs. 0)	−0.208	0.252	−0.748	0.332	0.423
Periodontitis recurrence (1 vs. 0)	0.407	0.238	−0.103	0.916	0.109

**Table 6:** Regression analysis with LDF (mm) from 1 year to 5 years post-operatively as a dependent variable. *R*^2^ = 0.7919.

Parameter	Regression Coefficient	Standard Error	Confidence Interval		*p*
			Lower	Upper	
Intercept					0.303
Sex (male vs. female)	−0.163	0.235	−0.666	0.341	0.500
Age	−0.190	0.146	−0.503	0.123	0.215
Surgical procedure (test vs. control)	−0.021	0.151	−0.345	0.303	0.891
Tooth type (molars vs. incisors, canines, and premolars)	−0.082	0.241	−0.600	0.435	0.738
Tooth position (upper vs. lower)	−0.031	0.195	−0.450	0.388	0.876
FMPS	−0.262	0.287	−0.877	0.354	0.378
FMBS	0.527	0.256	−0.022	1.077	0.059
PPD	0.097	0.238	−0.414	0.608	0.690
CAL	0.071	0.234	−0.431	0.572	0.767
RVG angle	0.047	0.177	−0.331	0.426	0.793
Patients’ compliance (1 vs. 0)	−0.363	0.184	−0.757	0.031	0.068
Periodontitis recurrence (1 vs. 0)	0.763	0.172	0.394	1.131	0.001 *

* statistically significant (*p* ≤ 0.05).

## Data Availability

The data presented in this study are available on request from the corresponding author.

## Abbreviations

The following abbreviations are used in this manuscript:

BD	Base of the Defect
CAL	Clinical Attachment Level
CAL-G	Clinical Attachment Level Gain
CEJ	Cementoenamel Junction
CI	Confidence Interval
DBBM	Deproteinized Bovine Bone Mineral
DBM	Demineralized Bone Matrix
DD	Radiographic Defect Depth
DFDBA	Demineralized Freeze-Dried Bone Allograft
FDBA	Freeze-Dried Bone Allograft
FMBS	Full-mouth Bleeding Score
FMPS	Full-mouth Plaque Score
FRSABG	Frozen Radiation-Sterilized Allogenic Bone Graft
GR	Gingival Recession
GTR	Guided Tissue Regeneration
LDF	Linear Defect Fill
MPPT	Modified Papilla Preservation Technique
n	Number of Defects
OFD	Open-Flap Debridement
PPD	Probing Pocket Depth
PPD-R	Probing Pocket Depth Reduction
PRF	Platelet-Rich Fibrin
RCT	Randomized Controlled Trial
SD	Standard Deviation
SPC	Supportive Periodontal Care
SPPF	Simplified Papilla Preservation Flap
%DF	Percentage Defect Fill
